# Delays and routes to diagnosis of neuroendocrine tumours

**DOI:** 10.1186/s12885-018-5057-3

**Published:** 2018-11-16

**Authors:** Ron Basuroy, Catherine Bouvier, John Keith Ramage, Maia Sissons, Raj Srirajaskanthan

**Affiliations:** 1 Department of Liver Sciences, Division of Transplantation Immunology & Mucosal Biology, King’s College London, London, UK; 2 Neuroendocrine Tumour Patient Foundation, Leamington Spa, UK; 3 ENETS Centre of Excellence, Neuroendocrine Tumour Unit, Kings College Hospital, London, UK; 4 Department of Gastroenterology, Kings College Hospital, London, SE5 9RS UK

**Keywords:** Symptoms, Neuroendocrine tumour, Gastrointestinal, Lung, Delay, Diagnosis and pancreas

## Abstract

**Background:**

Neuroendocrine tumours are uncommon tumours; there is often a long period between the onset of symptoms and diagnosis. This study aims to address the symptoms prior to diagnosis of people with known neuroendocrine tumours and also the involvement of healthcare providers prior to the diagnosis.

**Methods:**

A web based survey was designed to cover two broad areas of patient symptoms and healthcare interactions prior to diagnosis. This was tested and adapted by patient and clinician input prior to distribution via Survey Monkey.

**Results:**

The results demonstrated a median time from first symptom to diagnosis of 53.8 months. The most frequent initial symptoms were of pain, change in bowel habit and fatigue. 31% of respondents noted weight loss prior to diagnosis. 80% of respondents visited their GP regarding the symptoms a median of 11 times. 58% of respondents were referred to secondary care where they were seen a median 3 times. 30% presented acutely to A&E and this led to their diagnosis.

**Conclusion:**

In conclusion, there is a long time from onset of symptoms to diagnosis in all types of NETs. This is despite many respondents having alarm symptoms at diagnosis. Further education and awareness regarding malignancy may help with earlier diagnosis.

## Background

Neuroendocrine tumours (NETs) are rising in incidence and prevalence [1]. These tumours can arise in most organs of the body and can present with a multitude of symptoms [2]. They are regarded as rare cancers with a reported incidence of 5–7 per 100,000 population per year and estimated prevalence of 35 per 100,000 [1]. Symptoms leading to diagnosis are varied and dependent in part on the primary site of the tumour and also whether the tumours are functional in nature and causing a clinical syndrome such as carcinoid syndrome [3]. The majority of tumours are non-functional and consequently the presentation may be incidental or related to mass effect of tumour or metastatic disease [4]. The functional tumours can cause clinical syndromes; the most commonly recognised is carcinoid syndrome, though other symptoms/syndromes can occur, for example Zollinger Ellison syndrome in gastrinoma or Werner Morrison syndrome in VIP secreting tumours [5].

Anecdotally there is thought to be a delay in diagnosis due to the nature of the symptoms and patients being given an incorrect initial diagnosis [6, 7]. The duration of a delay in diagnosis is unclear but there are reports in the literature of delays in years prior to diagnosis. Patients commonly have diagnoses confused with other conditions such as dyspepsia or irritable bowel syndrome [8]. The incidence of misdiagnosis is unclear and the duration to diagnosis is also not well established.

There is minimal literature on the presenting symptoms of patients with NETs and the duration of symptoms prior to diagnosis [9]. It is also not clear how long symptomatic NET patients are investigated and managed before diagnosis by healthcare practitioners in both primary and secondary care, and whether this contributes to an avoidable delay.

The aims of this study were to understand the symptoms patients experience prior to diagnosis, the duration of these symptoms and their interaction with healthcare services prior to diagnosis. The survey was open to all patients with NETs to enable an overall view of symptoms prior to diagnosis and also assess whether time to diagnosis was different between different tumour sites.

## Methods

In collaboration with the NET patient foundation (NPF) we designed a web based survey. The NPF is a UK based neuroendocrine patient organization. The survey was created on SurveyMonkey and covered two broad areas of patient symptoms and healthcare interactions prior to diagnosis. No personal data questions, including contact details, were incorporated into the survey. Using the HRA decision aids tool an online form was completed, it confirmed that NHS REC approval was not required. The survey was entirely voluntary, and respondents were fully informed on the website about why the survey was being done.

We employed free text open questions at the start of the survey to enable a broad depth of data capture. Mandatory questions enabled us to filter responses to relevant sections and hence avoid respondent fatigue. Questions were developed using a multiple choice options, a 5 point scale or free text. The questions focusing on timeline and duration of symptoms were calculated using the baseline data of patients current age and the data at which they completed the survey.

The survey contained 130 questions and took around 30 min to complete. The set of questions were designed by the authors and clinical team at Kings College hospital Neuroendocrine Tumour Unit. The survey was then tested and adapted by trialing with patients and clinicians. The groups of questions included first and most troublesome symptoms, weight and appetite change, alarm signs, GP and hospital interactions, and diagnosis. Respondents were asked about change in weight and with weight loss of 4.5 kg or more categorized as relevant weight loss.

A link to the survey was posted on the NPF website and via their regular newsletter and Twitter update. In addition, the survey was distributed via clinical nurse specialists from NET centers around England using business cards containing the URL to access the survey. Following obtaining 300 responses the survey was closed, this was to ensure that the data would be sufficiently robust to describe patterns in symptoms and healthcare interactions. Data coding and analysis was performed with Microsoft® Excel® for Mac (2011). Free text response data was searched and categorised with key word searches. Descriptive statistics were used given the lack of counterfactual.

## Results

There was a total of 303 responses of which 229 completed the whole survey (75.6%). The mean time to complete the survey was 34 min. The majority of respondents were female patients (205/303, 67.65), including those who completed the whole survey (154/229, 67.2%). 76% of respondents (231/303) completed the ethnicity question (grouped as per UK Office for National Statistics) with the overwhelming majority describing themselves as of white ethnicity (94%). The mean age of respondents completing the survey was 55.7 years old with the mean ages for female and male respondents of 53.3 and 60.8 years old respectively. Respondents completed the survey on average 4 years after their initial NET diagnosis (mean age at diagnosis 51.6 years old). The interval between diagnosis and survey responses may lead to recall and reporting biases. The mean age at diagnosis was 51.6 years old for all respondents. Table 1. illustrates the number of respondents to the survey by primary site and also the average age of diagnosis.

**Table 1:** This demonstrates the mean age at diagnosis of the respondents by primary site. In addition, identifying the % of respondents over the age of 50 at time of first symptom

Type of NET	No.	Mean age at Diagnosis (years)	% with symptoms at diagnosis	Mean duration 1st symptom (range, months)	Mean Age at start of 1st symptom (years)	% aged over 50 at 1st symptom
Appendix	14	44.2	71%	46.8 (2–180)	41.7	29%
Lung	51	50.7	59%	67.7 (1.5–360)	46.2	54%
Not sure	20	47.5	70%	71.9 (2–264)	43.3	55%
Ovary	2	44.3	50%	5.00 (4–6)	43.8	0%
Pancreas	64	49.2	73%	39.1 (0–240)	46.6	42%
Rectal	5	45.4	60%	41.1 (1–120)	42.0	60%
Renal/Kidney	1	48.0	100%	–	–	–
Small Bowel	99	55.2	83%	60.1 (0–300)	50.8	69%
Stomach/gastric	14	55.1	71%	38.5 (1–144)	53.0	71%
Unknown Primary	33	52.9	70%	43.4 (1.5–204)	50.4	55%
Total/(Mean)	303	(51.6)	(73%)	(53.8)	(48.1)	(56%)

The mean duration of the first symptom prior to diagnosis for respondents was 53.8 months overall, 60.1 months for small bowel NETs (sbNETs), 39.1 months for pancreatic NETs (pNETs) and 67.7 months for lung NETs. The mean age at onset of first symptom was 48.1 years for all respondents and 50.8, 46.6 and 46.2 years for small bowel, pancreatic and lung NETs respectively. Over half of all respondents (56%) were aged over 50 years old at the time of developing their first NET symptom.

### Symptoms prior to diagnosis

80% of respondents reported that they had symptoms prior to diagnosis (243/303). Most of these respondents reported that their symptoms led to the NET diagnosis (73%, 221/303), in particular those with pNETs (73%, 47/64) and small bowel NETs (83%, 82/99). Over half of respondents reported that their symptoms led to a scan (124/221, 56%) and a subsequent NET diagnosis. 66% of pNET respondents (31/47) and 57% of sbNET respondents (47/82) had symptoms that led to a scan and diagnosis.

### First symptom

**Fig. 1:**
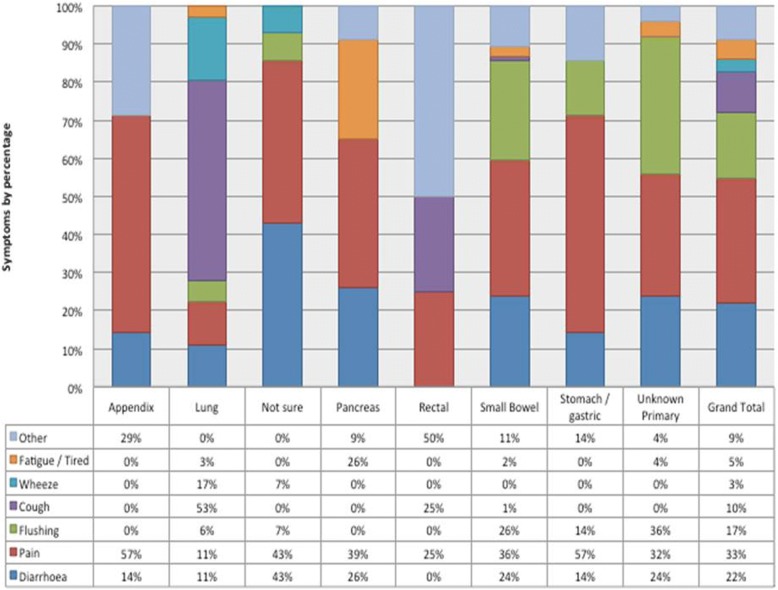
Illustrates the frequency of the seven main primary symptoms reported by respondents dependent on the site of primary tumour

81% of symptomatic respondents (180/243) described their first symptom in free text that was categorized into seven main areas; diarrhoea, pain, flushing, cough, wheeze, tiredness/fatigue and other. The majority of respondents described a single symptom (70%, 126/180) while the remainder described two or more symptoms in free text from the seven main areas. The most frequently described first symptom prior to diagnosis was pain (33%) followed by diarrhoea (22%), flushing (17%) and cough (10%). Respondents with small bowel NETs described pain (36%), flushing (26%) and diarrhoea (24%) as the most common first symptoms. Respondents with pancreatic NETs described pain (39%), diarrhoea (26%) and fatigue or tiredness (26%) as the most common first symptoms. Respondents with lung NETs described cough (53%), wheeze (17%), pain (11%) and diarrhoea (11%) as the most common first symptoms. The symptoms appear related to the primary site for example abdominal pain being frequently reported with gastrointestinal NETs. Figure 1. lists the primary symptoms commonly associated with the tumour site. Almost all respondents who reported symptoms prior to diagnosis (99%, 240/243) graded the severity of their symptoms on a 5-point scale (very mild, mild, moderate, severe, very severe). 63% of all those who responded reported their first symptom before diagnosis was severe or very severe (151/240). Table 2. describes the severity of the initial symptom based on tumour site.

**Table 2:** Describes the severity of the primary symptom as reported by respondents from different primary sites

Severity	Appendix	Lung	Not sure	Ovary	Pancreas	Rectal	Small Bowel	Stomach/gastric	Unknown Primary	Overall
Very mild	10%	5%	0%	0%	0%	20%	2%	0%	0%	3%
Mild	20%	11%	0%	0%	12%	20%	5%	22%	13%	9%
Moderate	30%	26%	33%	0%	24%	60%	24%	22%	22%	25%
Severe	40%	32%	53%	100%	46%	0%	39%	44%	43%	40%
Very severe	0%	26%	13%	0%	18%	0%	31%	11%	22%	23%
Responses	10	38	15	2	50	5	88	9	23	240

### Other severe symptom

Over half of all symptomatic respondents (133/243, 55%) reported in free text that they had a different more severe symptom prior to diagnosis from their initial symptom. 59% of these respondents (78/133) described this additional more severe symptom in free text that was categorized into seven main areas; diarrhoea, pain, flushing, cough, wheeze, tiredness/fatigue and other. There was limited response data for NETs beyond those with pancreatic, small bowel and lung NETs. 78% of all those who responded reported their severest symptom as severe or very severe (103/133). 80% of sbNET respondents graded their most severe symptom as severe or very severe compared with 90% of pNET and 60% of lung NET respondents.

### Weight and appetite change

42% (126/303) of respondents reported no change to weight prior to their diagnosis, 31% reported weight loss (93/303) and 19% reported weight gain (58/303). There were similar proportions of sbNET (37/99, 37%) and pNET (19/64, 30%) respondents reporting weight loss, while there was a lower proportion for lung NETs respondents (10/51, 20%).

55% (168/303) of respondents reported no change to their appetite prior to diagnosis, 25% reported appetite loss (77/303) and 10% reported appetite gain (31/303). There were similar proportions of sbNET (31/99, 31%) and pNET (14/64, 22%) respondents reporting appetite loss, while there was a lower proportion for lung NETs respondents (9/51, 18%). However, the majority of sbNET, pNET and lung NET respondents reported no change to their appetite prior to diagnosis; 61% (60/99), 53% (34/64) and 65% (33/51) respectively. Table 3. describes the changes in weight and appetite in detail for respondents of each tumour site.

**Table 3:** This identifies the change in weight and appetite reported by respondents by primary site

		Weight				Appetite			
Row Labels	Respondents	Loss	Gain	No change	Unsure	Decrease	Increase	No change	Unsure
Appendix	14	21%	14%	36%	29%	21%	7%	50%	21%
Lung	51	20%	33%	37%	10%	18%	10%	65%	8%
Not sure	20	35%	25%	15%	25%	25%	20%	15%	40%
Ovary	2	100%	0%	0%	0%	100%	0%	0%	0%
Pancreas	64	30%	22%	44%	5%	22%	20%	53%	5%
Rectal	5	0%	40%	60%	0%	0%	20%	80%	0%
Renal/Kidney	1	0%	0%	100%	0%	0%	100%	0%	0%
Small Bowel	99	37%	15%	42%	5%	31%	3%	61%	5%
Stomach/gastric	14	36%	7%	57%	0%	29%	0%	57%	14%
Unknown Primary	33	30%	6%	52%	12%	27%	9%	58%	6%
Overall		31%	19%	42%	9%	25%	10%	55%	9%
Respondents	303	93	58	126	26	77	31	168	27

The three most common primary sites are highlighted in yellow

The mean weight change for sbNET and pNET respondents was a loss of − 4.1 and − 0.4 kg respectively. 60% of sbNET and 45% of pNETs reported greater than 4.5 kg weight loss. Weight change was reported over a mean duration of 24 months for respondents with a similar duration for sbNETs (26 months) but a shorter duration for pNETs (15 months). Cohorts of respondents who also reported a loss of appetite (number, %) had marked weight loss; − 9.8 kg overall (77/303, 25%), − 11.7 kg sbNETs (31/99, 31%), − 6.6 kg pNETs (14/64, 22%) and − 10.8 kg lung NETs (9/51, 18%).

### Abdominal and back pain

69% of respondents (175/252, excluding those with lung NETs) reported abdominal or back pain prior to diagnosis. Table 4, details the presence of abdominal and or back pain reported by respondents from different tumour sites. As expected 93% of appendiceal NETs described pain, however, this was likely from the appendicitis that led to their presentation rather than the tumour. However, small bowel NET (81%, 80/99) respondents reported pain more than pNET (55%, 35/64) respondents prior to the diagnosis. sbNET respondents described the pain as starting in the periumbilical (26%) or pelvic area (24%) before becoming more generalized to the abdominal midline and lower abdomen (62%) prior to diagnosis. pNET respondents described the pain as starting in the epigastric (39%) or upper right abdomen area (10%) before becoming more diffuse in it’s location prior to diagnosis. There was no differentiating characteristic to the pain experienced by sbNET and pNET respondents, or association with other intestinal symptoms (bowel frequency, stool consistency, partial obstruction). The frequency of the pain was similar for both sbNETs and pNETs, occurring a few times a month or greater than once a week. sbNET respondents described a more severe pain profile than pNET respondents.

**Table 4:** This table documents the incidence of abdominal pain and/ or as described by respondents dependent on the primary site of the tumour

Row Labels	Respondents	Yes	No	Not sure /−
Appendix	14	93%	0%	7%
Not sure	20	50%	10%	40%
Ovary	2	50%	50%	0%
Pancreas	64	55%	39%	6%
Rectal	5	60%	20%	20%
Renal / Kidney	1	0%	0%	100%
Small Bowel	99	81%	15%	4%
Stomach / gastric	14	71%	21%	7%
Unknown Primary	33	70%	21%	9%
Overall	100%	69%	21%	9%
Respondents	252	175	54	74

### Bowel symptoms

Half of respondents (125/252, excluding lung NETs) reported problems with their bowels prior to diagnosis. The proportion experiencing bowel problems was greater for sbNETs (64%, 63/99) than for pNETs (36%, 23/64). Three quarters of respondents (75%, 62/83) experienced problems with loose, mushy or watery stools prior to diagnosis. 46% of sbNET respondents (21/46) described the problem with loose bowels as severe or very severe. 60% of small bowel NET patients reported that ¾ of their stools were loose prior to diagnosis. 66% of sbNET respondents (57/87) reported some degree of urgency to open their bowels and 78% (68/87) reported some degree of bloating.

### Other symptoms

67% of respondents (95/141, excluding those with lung NETs) reported flushing prior to diagnosis. Small bowel NET (83%, 72/87) respondents reported flushing more than pNET (43%, 23/54) respondents prior to the diagnosis. Almost a third of sbNET respondents described the flushing as severe or very severe (31%, 22/72). Alcohol and large meals were reported by sbNET respondents to make the flushing worse. 60% of respondents (84/141, excluding those with lung NETs) reported anxiety prior to diagnosis. Small bowel NET (66%, 57/87) respondents reported anxiety more than pNET (50%, 27/54) respondents prior to the diagnosis. A small proportion of sbNET respondents described the anxiety as severe or very severe (16%, 9/57). Respondents reported nausea in 45% and vomiting in 36% of cases (65/146 and 52/146 respectively, excluding those with lung NETs) prior to diagnosis. 35% of respondents (49/139, excluding those with lung NETs) reported problems with breathing prior to diagnosis. The respondent data available limited further analysis of these symptoms.

### Accessing healthcare services and diagnosis

The majority of respondents (80%) reported that they saw their GP with symptoms prior to their NET diagnosis. Respondents reported they saw their GP over a mean period of 37 months and a mean of 11 interactions. There was no marked difference in the mean period and interactions in primary care for respondents. Table 5. details the interaction of respondents with primary care. The duration of recurrent attendance to primary care implies that symptoms had not improved. It seems that initial incorrect diagnosis was given to patients based on the high number of respondents reporting a diagnosis of functional bowel syndrome, such as irritable bowel syndrome, or dyspepsia. Table 6. outlines the initial diagnosis given to respondents in primary or secondary care.

**Table 5:** This shows the number of respondents that attended their primary care provider with symptoms from the NET

		Primary Care			Secondary Care		
Site of NET	Respondents	% seen by GP	Mean No. times seen by GP	Mean time GP investigated (months)	% seen in clinic	Mean No. times seen in clinic	Mean time clinic investigated (months)
Lung	51	73%	14	44	55%	3	4
Pancreas	52	73%	8	33	50%	4	21
Small Bowel	86	86%	10	40	62%	3	11
All/(mean)	257	(80%)	(11)	(37)	(58%)	(3)	(17)

The number of visits related to the GP and also secondary care if referred via their GP. The final column encompasses all respondents that completed this part of the questionnaire and not separated by tumour site

**Table 6:** This table lists the commonly made initial diagnosis in primary and secondary care when respondents attended with the symptoms related to their NET

Potential cause	Primary Care			Secondary Care		
	Pancreas	Small Bowel	Grand Total	Pancreas	Small Bowel	Grand Total
IBS	16%	29%	24%	5%	12%	9%
Dyspepsia	9%	16%	13%	3%	3%	3%
Depression	5%	3%	4%	2%	1%	1%
UTI	5%	3%	4%	0%	0%	0%
Constipation	3%	5%	4%	2%	1%	1%
Gall Stones	8%	12%	10%	3%	5%	4%
Menopause	2%	11%	7%	0%	3%	2%
Chest infection	2%	2%	2%	0%	0%	0%
Haemorrhoids	0%	0%	0%	0%	0%	0%
Kidney stones	5%	3%	4%	3%	1%	2%
Anaemia	5%	5%	5%	2%	0%	1%
Crohn’s	0%	2%	1%	2%	1%	1%
NET	0%	2%	1%	20%	22%	21%
Ulcerative colitis	5%	4%	4%	0%	1%	1%
Cancer	3%	4%	4%	17%	14%	15%
They were not sure	9%	19%	15%	6%	15%	12%
Not sure	8%	8%	8%	6%	5%	6%
Other	22%	25%	24%	23%	23%	23%
Respondents	64	99	163	64	99	163

The percentage is derived from the initial diagnosis given to patients by the total number of patients. For example 10 of 64 patients (16%) with pNETs were initially given a diagnosis of IBS. *Abbreviations*: *IBS* irritable bowel syndrome, *UTI* urinary tract infection, *NET* neuroendocrine tumour

Over half of respondents (58%, 122/210) reported that they first interacted with secondary care from a GP referral to a local hospital clinic. Almost a third of respondents (31%, 66/210) reported that their first secondary care interaction was via an unplanned emergency admission from A&E (see Table 7). Again there was no marked difference in the routes that sbNET and pNET respondents first interacted with secondary care. 43% of pNET and sbNET respondents reported that they were investigated in gastroenterology clinics prior to diagnosis with others mainly investigated in surgical or oncology clinics. Respondents reported that they were investigated in clinic for a mean period of 17 months over a mean of 3 occasions. There is likely to be overlap in primary and secondary care management (37 and 17 months respectively) given respondents also reported being symptomatic for similar period of time (mean 53.8 months) and the likelihood of a staggered presentation to first access healthcare. However, it was not possible to quantify the overlap in primary and secondary care given the questionnaire design.

**Table 7:** This table demonstrates the number of patients presenting to A&E either directly or following referral via GP

Site of NET	Emergency admission from A&E	Via a GP referral as an emergency admission to the local hospital	Via a GP referral to a clinic at the local hospital	Numbers
Appendix	30%	40%	30%	10
Lung	30%	18%	52%	33
Not sure	50%	20%	30%	10
Pancreas	40%	4%	56%	45
Rectal	0%	0%	100%	3
Renal/Kidney	0%	0%	100%	1
Small Bowel	32%	5%	63%	76
Stomach/gastric	22%	11%	67%	9
Unknown Primary	17%	13%	70%	23
Overall %	31%	10%	58%	100%
Numbers	66	22	122	210

In addition, % of patients that are referred to secondary care by primary tumour site

## Discussion

The survey is one of the most detailed in terms of exploring symptoms and access to healthcare prior to diagnosis of patients with NETs. The majority of respondents had, as expected, small bowel, pancreatic or lung NETs. However, responses were received from respondents with tumours arising from a number of different primary sites. The mean age of the respondents to the survey was somewhat younger than the mean age of diagnosis of NET. This is in part related to the inherent bias in asking patients with NETs to only complete an online survey.

The time from first symptom to diagnosis was 53.8 months, which is a very long time especially when considering the number of respondents that regarded their primary symptom as being severe or very severe in nature. A significant delay is likely to be occurring due to incorrect initial diagnosis. Commonly, functional bowel disorders were the initial diagnosis in a number of cases.

There is a scarcity of data on delays and routes to diagnosis for patients with neuroendocrine tumours. In 2014, the International Neuroendocrine Cancer Alliance (INCA) commissioned a general global survey of patients with NETS. Part of this questionnaire addressed routes and time to diagnosis. Interestingly, they reported a mean patient reported time from first symptom to diagnosis of 52 months; with 29% of patients requiring greater than 5 years for a NET diagnosis [6]. A subgroup of these respondents from USA was further analysed and this confirmed that patient reported time from first symptom to onset of clinical diagnosis of 59 months [10]. This data is similar to what we have demonstrated in this study and indicates that is different models of healthcare delivery there appear to be a similar delay in diagnosis.

Seventy-four (24%) respondents had lost greater than 4.5 kg in weight and this should lead to investigation as to the cause of this weight loss. Furthermore, over half of respondents were aged over 50 years old and had red flag symptoms at the time of presentation including weight loss and reduced appetite. Whilst a fraction of patients met the criteria for conditions such as irritable bowel syndrome, the majority has an age of onset of symptoms over 50 and other alarm symptoms that should warrant investigation prior to assuming a diagnosis of a functional syndrome such as dyspepsia.

The survey has clearly demonstrated that the majority of respondents with NETs do not present with typical symptoms. For example, carcinoid syndrome symptoms were not being floridly reported by those with small bowel NETs. Therefore, the diagnosis of NETs will require investigation of vague nonspecific often abdominal or gastrointestinal related symptoms. Therefore, the role of cross sectional imaging is important to help expedite this diagnosis.

Reduced health-seeking behaviour in UK patients has been highlighted as a factor in later stage disease at presentation and consequent worse outcomes than other developed countries [11]. The public health messaging around cancer symptomatology and the introduction of screening programmes, such as the bowel cancer screening programme, can help improve outcomes but have no clear secondary benefit for other malignancies like NET [12]. Patients with symptoms like diarrhoea and pain may be investigated with simple and accessible diagnostics, such as abdominal ultrasound, endoscopy and blood testing, that maybe falsely reassuring given the low sensitivity for early NET disease. Cross-sectional modalities like CT and/or MRI may reveal earlier stage disease, particularly when used in patients who are 50 years old with new symptoms.

This survey does clearly demonstrate that there is no reduced health seeking behavior from these respondents with 80% of respondents visiting their GP on average 11 times related to the symptoms from NET prior to diagnosis. This suggests that it is not healthcare avoidance by the public, but lack of onward referral or appropriate investigations in the primary and secondary care setting that seems to be contributing to the delay in diagnosis.

The survey was limited by its retrospective nature and inclusion of all historical NET patients. There was an average of four years from diagnosis to completing the survey that could significantly bias a patient’s recall. The patients’ perception of their initial symptoms being related to the tumour needs to be interpreted with caution in some cases. For example, 25% of participants with rectal NETs reported cough as their first symptom, which seems unlikely. In most cases however, the primary symptoms would fit with the underlying type of NET. For example, respondents with small bowel NETs described pain (36%), flushing (26%) and diarrhoea (24%) as the most common first symptoms. However, events and symptomatology that occurred well after diagnosis, including post-surgical symptoms, could easily be mixed with those occurring prior to diagnosis. The survey was predominantly accessed via the NET patient foundation’s digital promotional channels that could represent a self-selecting informed and proactive cohort of NET patients. The responses may be not representative of the majority of NET patients in the UK who may have different symptom, disease and healthcare experiences. The mean age at diagnosis in the cohort that responded to the study is younger than would be expected based on epidemiological data. Therefore, suggesting a selective group that use online platforms or have social media accounts.

## Conclusion

In summary, this survey demonstrates a mean time of 53.8 months from onset of symptoms to diagnosis. The primary symptoms from respondents appear linked to the primary site of the tumour and associated weight loss is a common finding. Respondents are seeking healthcare but are often misdiagnosed with functional gastrointestinal disorders. Further education regarding investigation of malignancy in patients over 50 and greater use of cross sectional imaging for patients with alarm symptoms over 50 may help shorten the time to diagnosis of NETs.
